
JOURNAL INFORMATION
==============================
NLM Title Abbreviation: Alcohol Res Health
Journal ID: Alcohol Res Health
NLM Journal ID: 100900708
Journal ID: ARH

Alcohol Research & Health

ISSN: 1535-7414
EISSN: 1930-0573
Publisher: National Institute on Alcohol Abuse and Alcoholism

ARTICLE INFORMATION
==============================
PMCID: PMC3860430
Article ID: arh-30-1-32
Article version: 1
Subjects: Glossary

Glossary

Print publication date: 2007
Volume: 30
Issue: 1
First page: 32
Last page: 36
Copyright year: 2007
License: Unless otherwise noted in the text, all material appearing in this journal is in the public domain and may be reproduced without permission. Citation of the source is appreciated.
License URL: https://creativecommons.org/publicdomain/mark/1.0/

Comment in: The Genetics of Alcohol Metabolism: Role of Alcohol Dehydrogenase and Aldehyde Dehydrogenase Variants 30 1 2007 5 13 Alcohol Research & Health PMC3860432 17718394
Comment in: Alcohol Metabolism and Cancer Risk 30 1 2007 38 47 Alcohol Research & Health PMC3860434 17718399

==============================
Acetaldehyde A toxic byproduct of alcohol metabolism.

Acetate A compound produced from the metabolism of acetaldehyde.

Adduct Product of the addition of one compound (e.g., acetaldehyde) to another compound (e.g., DNA).

Adenoma A usually benign tumor of tissue lining the internal and external organs of the body (i.e., epithelial tissue) in which the tumor cells are arranged in a gland-like structure.

Adenomatous polyps A tissue mass that bulges outward from the normal surface and which consists of benign tumor tissue; found, for example, in the colon.

Adenosine triphosphate (ATP) A molecule, generated largely in the mitochondria, that provides the energy needed for many key metabolic reactions.

Affinity Measure of the strength with which an enzyme (e.g., alcohol dehydrogenase [ADH]) interacts with its target molecule (e.g., ethanol); an enzyme variant with a higher affinity can interact with its target at lower concentrations of the target molecule than an enzyme variant with a lower affinity.

Alcohol dehydrogenase (ADH) An enzyme that breaks down alcohol by oxidation, converting it to acetaldehyde. (See cytochrome P450.)

Aldehyde dehydrogenase (ALDH) An enzyme that converts acetaldehyde to acetate.

Allele One of two or more variants of a certain gene.

Amine A type of organic compound that contains nitrogen as a central atom.

Amino acids The principal building blocks of proteins and enzymes.

Amino group A group of atoms found in all amines and amino acids.

Antibody A protein produced by certain immune cells that recognizes and binds to foreign proteins, leading to the destruction of those proteins.

Antibody-dependent cell-mediated cytotoxicity (ADCC) A mechanism of cell-mediated immunity whereby certain immune cells actively break up a target cell that has been bound by specific antibodies.

Antioxidant A substance, such as glutathione, vitamin E, or an enzyme, that inhibits oxidation and that scavenges free radicals and protects the cell against damage caused by these radicals.

Apoptosis Cell death in which the affected cell participates by activating a cascade of biochemical reactions that lead to death; also known as programmed cell death or cell suicide.

Aromatic amino acids A class of amino acids, including phenylalanine and tryptophan, in which some of the constituent atoms form a ring.

Carcinogenesis The process of initiating and promoting cancer.

Carcinoma A malignant tumor of the epithelial tissue that tends to invade surrounding tissue and metastasize to other regions of the body.

Case–control study An epidemiologic approach in which previously existing cases of a condition (e.g., a type of cancer) are compared with a control group of people who have similar characteristics (e.g., gender, age, and alcohol use history) but have not developed the condition under investigation; the two groups are compared to determine which factor (e.g., ALD allele) may account for the increased disease incidence in the case group.

Catalase An enzyme that catalyzes the decomposition of hydrogen peroxide into water and oxygen.

Central vein Blood vessel located in the center of each liver lobule through which cleansed blood exits the lobule and which feeds into the hepatic vein; also called hepatic venule.

Coenzyme A nonprotein substance that combines with an enzyme to form a complete, functional complex.

Cohort study An epidemiologic approach in which a group of people who share a common characteristic (e.g., who were all born in the same town or who all entered the an alcoholism treatment program) are followed to determine which of them develop a certain condition (e.g., cancer).

Cytochrome P450 A family of cytochromes, one of which (CYP2E1) can oxidize alcohol to form acetaldehyde; high alcohol levels stimulate CYP2E1 activity.

Cytochromes Specialized enzymes within mitochondria and other cell structures. Different cytochromes play important roles in metabolizing toxic substances, drugs, and other chemicals, as well as in producing adenosine triphosphate (ATP).

Cytokines A family of molecules, produced primarily by cells of the immune system, that regulate cellular interactions and other functions. Many cytokines play important roles in initiating and regulating inflammation.

Cytoplasm The substance filling the cell, including the cytosol as well as mitochondria, endoplasmic reticulum, and other cell structures (organelles) but excluding the nucleus.

Cytosol The fluid portion of the cytoplasm.

Dimer Compound formed by the combination of two simpler molecules (subunits) that normally are not functional by themselves.

Electron A subatomic particle with a negative charge.

Endocytosis Mechanism by which specific molecules are ingested into the cell.

Endoplasmic reticulum A system of folded membranes that loop back and forth, spreading throughout the cytoplasm and providing a large surface area for cell reactions.

Endothelial cells Type of cell lining the body cavities and blood vessels; control the passage of materials and the transit of white blood cells into and out of the bloodstream.

Enzyme A substance, usually a protein, that directs and accelerates chemical reactions in the body but does not itself undergo permanent change.

Ester A compound formed from an acid and an alcohol.

Exon Part of a gene that encodes a section of the mature messenger RNA (mRNA) by splicing and therefore is converted into a protein.

Expression (i.e., gene expression) The process by which the genetic information encoded in a gene’s DNA sequence is converted into a functional protein.

Fatty acids A major component of fats that is used by the body for energy and tissue development.

Fibrosis The formation of scar tissue.

Free radicals Highly reactive molecular fragments that frequently contain oxygen. (See reactive oxygen species [ROS].)

Genotype The complete genetic makeup of an organism determined by the particular combination of alleles for all genes.

Haplotype Set of variations (e.g, single-nucleotide polymorphisms [SNPs]) that are inherited together.

Hepatic vein A large vessel that receives blood after it has passed through the central veins of the liver lobules.

Hepatocytes The principal cells of the liver, which carry out most of the liver’s metabolic activities.

Heterodimer Dimer made up of two different subunits.

Heterozygous Carrying two different alleles of a given gene.

Homodimer Dimer made up of two identical subunits.

Homozygous Carrying two copies of the same allele of a given gene.

Hyperlipidemia Excess fat in the blood.

Hyperuricemia Excess uric acid in the blood.

Hypoxia Lower-than-normal levels of oxygen.

In vitro Latin term meaning “in glass”; refers to experiments that are not conducted in an intact organism but in a test tube.

Intron DNA sequence located between two exons in a gene; although it is transcribed during gene expression, it is removed from the final messenger RNA (mRNA) by splicing and therefore is not converted into protein.

Isozymes/Isoenzymes Enzymes that differ in amino acid sequence but catalyze the same chemical reaction.

Ketosis Abnormal accumulation in the body of ketones, which are end products of fatty acid metabolism. Ketosis occurs when the body cannot metabolize sufficient carbohydrates to generate the energy needed (e.g., in patients with diabetes or during starvation).

Kinetic properties Variables that describe the activity of an enzyme, such as its turnover rate (i.e., the number of reactions it can perform during 1 minute) or its binding constant (Km), which describes the enzyme’s affinity to its target molecule.

Km A measurement used to describe the activity of an enzyme. It describes the concentration of the enzyme’s substrate at which the enzyme works at 50 percent capacity.

Kupffer cells Specialized immune cells in the liver that filter bacteria and other foreign substances from the blood and produce antibodies and cytokines. (See also sinusoids.)

Lactic acidosis A condition characterized by the accumulation of lactic acid in bodily tissues.

Linkage disequilibrium (LD) Phenomenon in which alleles at two or more sites on a chromosome are not randomly associated—that is, a particular allele at one site may almost always occur on chromosomes with a specific allele at another site. For example, there are two coding single-nucleotide polymorphisms (SNPs) in ADH1C, but instead of all four possible combinations of the two amino acids (as would be expected if the SNPs were randomly associated), only two forms are commonly found.

Linkage study The comparison of two groups of subjects (e.g., people with and without a given disease) to evaluate association between an allele and a phenotype (e.g., a disease).

Lipids Fatty substances, including simple fats, their major components (i.e., fatty acids), and various fat-soluble substances (e.g., cholesterol).

Lipid peroxidation The sequential breakdown of fatty substances in cells by chemical oxidation, leading eventually to the destruction of membranes within and surrounding the cell.

Lobule A cylindrical structure about 2 millimeters in diameter that is the basic functional unit of the liver. The liver can be composed of up to 100,000 lobules.

Macrophage A type of immune cell that ingests foreign particles and microorganisms and synthesizes proteins and other substances important in inflammatory responses, including cytokines. Macrophages that reside in the liver are called Kupffer cells.

messenger RNA (mRNA) Intermediary molecule generated during the process of converting the genetic information encoded in the DNA into protein products.

Metabolism The totality of chemical reactions occurring in a cell, an organ, or the body. The term sometimes is applied more narrowly to the breakdown of a particular substance (e.g., alcohol) by specific enzymes.

Microsomes Small vesicles derived from fragmented endoplasmic reticulum produced when tissues such as liver are mechanically broken (homogenized). Microsomes contain the cell’s cytochrome P450 (CYP) enzymes, involved in oxidative metabolism.

Microsomal ethanol-oxidizing system An enzyme system involving cytochrome P450 that breaks down alcohol and generates toxic products, such as acetaldehyde and reactive oxygen species (ROS).

Microtubules Any of the minute tubules in cell cytoplasm that are composed of the protein tubulin and form important structural components.

Mitochondria Structures within cells that generate most of the cells’ energy through the production of adenosine triphosphate (ATP).

Mitochondrial electron transport system see Respiratory chain.

Morphologic Pertaining to the physical shape and size of an organ, tissue, or cell.

Mucosa Mucous membrane; a thin sheet of tissue that lines cavities or canals of the body that open to the outside (e.g., the aerodigestive tract) and which, among other functions, secretes mucus.

Mutagenic Causing a genetic mutation or increase in mutation rate.

Mutation A change in the genetic material that can occur either spontaneously or can be induced by a chemical or other process.

Nicotinamide adenine dinucleotide (NAD+) NAD is a molecule that can bind with hydrogen atoms and become reduced NAD, or NADH; it acts as a coenyzme that interacts with numerous enyzmes (including alcohol dehydrogenase [ADH]). NAD and NADH serve as a hydrogen acceptor and donor, respectively, during enzymatic reactions, thereby helping to maintain balance between oxidation and reduction in the cell.

Nucleotide Building block of DNA and other related molecules.

Odds ratio The ratio of the odds of an event (e.g., development of alcoholism) occurring in one group of individuals (e.g., people with the ALDH2*2 allele) versus the odds of that event occurring in another group (e.g., people with the ALDH2*1 allele); an odds ratio <1.0 indicates that the first group has a reduced risk of the event, whereas an odds ratio >1 indicates that the first group has an increased risk of the event compared with the second group.

Oncogene A potentially cancer-inducing gene that under normal conditions plays a role in growth and proliferation of cells but which, when activated in some way, may cause the cell to multiply uncontrollably.

Oxidation A chemical reaction that results in a loss of electrons by a substance and which usually involves removing a hydrogen atom from a molecule or adding oxygen to it, or both. (See reduction.)

Oxidative stress An imbalance between oxidants (e.g., free radicals) and antioxidants that can lead to excessive oxidation and cell damage.

Pancreatitis An acute or chronic inflammation of the pancreas.

Perivenous Referring to the region of a liver lobule surrounding the central vein.

Peroxisomes A cytoplasmic cell organelle containing enzymes that act in the production and decomposition of hydrogen peroxide.

Phospholipid A lipid that contains a phosphate group.

Polyamine Any compound that contains two or more amine (NH2) chemical groups; many polyamines occur naturally in the tissues, particularly in rapidly growing tissues.

Polymorphism Existence of a gene in several allelic forms.

Proliferation The growth and reproduction of cells.

Promoter Set of DNA elements that regulate in which cell, at what time, and in what amount a gene is expressed and which specifies the transcription start site.

Proteins Molecules composed of chains of amino acids linked together. Proteins help maintain the cell’s structure and participate in many biological functions, including the regulation of metabolic reactions. The shape and function of a protein is determined by the sequence of its amino acids.

Reactive oxygen species (ROS) Highly reactive oxygen-containing free radicals that are generated during oxidative metabolism. ROS can react with and damage lipids, proteins, and DNA in cells, causing oxidative stress. Common ROS include hydrogen peroxide, superoxide radicals, and hydroxyl radicals.

Receptor A protein on the surface of a cell that recognizes and binds to chemical messengers.

Recombination Rearrangement of genetic material during the production of the germ cells that results in a unique combination of genes in each individual; appears more commonly to occur at certain sites on the DNA (hot spots) than would be expected by chance alone.

Redox/Redox state Shorthand for reduction/oxidation reactions. The term redox state is often used to describe the balance of NAD+ and NADH in a biological system such as a cell or organ. An abnormal redox state can develop in a variety of deleterious situations.

Reduction The reverse of oxidation, reduction is a chemical reaction that results in a gain of electrons by a substance and which usually involves removing an oxygen atom from a molecule, or adding hydrogen to it, or both.

Relative risk The ratio of the frequency of a certain disorders (e.g., cancer) in groups exposed to a certain risk factor (e.g., heavy alcohol consumption) and in groups not exposed to the risk factor.

Respiratory chain The electron transport system located in the mitochondria, in which electrons released by NADH are passed on to a series of other molecules that first accept the electrons and then pass them on to the next molecule in the chain. The electrons ultimately are transferred to oxygen to generate water. These successive reactions provide enough energy to drive the synthesis of adenosine triphosphate (ATP) molecules.

Retinol Vitamin A.

Salivary gland One of three pairs of glands located around the mouth that secrete saliva into the mouth.

Single-nucleotide polymorphism (SNP) Genetic variation that results from the exchange of only a single nucleotide.

Sinusoids Channels in a liver lobule that bring blood and nutrients to the hepatocytes, similar to capillaries in other organs. Sinusoids are lined with endothelial cells and Kupffer cells.

Stellate cell A star-shaped liver cell that serves as the primary storage site for vitamin A compounds and fat molecules; activation of stellate cells plays a central role in the development of fibrosis.

Substrate A substance upon which an enzyme acts.

Superoxide A destructive reactive oxygen species (ROS) produced as a byproduct of some oxidation reactions.

Synergistic effects The combined effect of two factors or drugs that is greater than the sum of the effects of both factors individually.

Transcription Process by which the genetic information contained in the genes on the DNA is copied into an intermediary molecule (messenger RNA [mRNA]) that then serves as the template for translation.

Translation Process during which a protein is generated from amino acids based on the information carried in the messenger RNA (mRNA).

Tumor necrosis factor alpha (TNF-α) A type of cytokine that promotes inflammatory responses, stimulates neutrophils and macrophages, induces fever, and induces macrophages to produce cytokines.

Tumor suppressor gene A gene whose product normally serves to control cell growth and which, when inactivated, may cause the cell to multiply uncontrollably (also see oncogene).

